# Portomesenteric venous thrombosis in a postmenopausal female with testosterone implant: a case report

**DOI:** 10.1186/s13256-021-02805-6

**Published:** 2021-05-19

**Authors:** Monica Zanconato Campitruz, Luis T. Ortiz-Figueroa, Edgardo Santiago

**Affiliations:** 1 Hoboken University Medical Center, 308 Willow Avenue, Hoboken, NJ 07030 USA; 2 Penn State Health Milton S. Hershey Medical Center, 500 University Drive, Hershey, PA 17033 USA; 3 University of Puerto Rico Medical Sciences Campus, Paseo Dr. José Celso Barbosa, San Juan, PR 00921 USA; 4 West New York, USA

**Keywords:** Portal vein thrombosis, Superior mesenteric vein thrombosis, Prothrombotic states, Testosterone implant, Hormone replacement therapy

## Abstract

**Background:**

Acute portal vein thrombosis is a rare medical event usually seen in liver disease, but it can also occur due to any inherited or acquired procoagulable state that triggers venous occlusion. Hormonal therapies have been associated with an increased risk of prothrombotic states. This case report documents a portomesenteric venous thrombosis in a postmenopausal woman with testosterone implant for the treatment of hypoactive sexual desire and discusses the importance of identifying hypercoagulable risk factors before initiating hormone replacement therapy. We want to improve the awareness of an unusual medical complication associated with hormone replacement therapy and shed light on how testosterone implants could facilitate a thrombotic event related to other risk factors such as obesity and chronic hypoxic states, as well as the importance of differential diagnosis in the evaluation of postmenopausal women on testosterone replacement therapy presenting with acute abdominal pain.

**Case presentation:**

A 55-year-old obese postmenopausal Hispanic female with medical history of chronic obstructive pulmonary disease presents with intractable abdominal pain, is found to have elevated hemoglobin and hematocrit, and an abdominopelvic computed tomography scan revealing portal and superior mesenteric vein thrombosis. Further evaluation excluded inherited and acquired thrombophilia but revealed elevated testosterone levels. The patient was treated with anticoagulation, which resulted in recanalization of the portal and superior mesenteric veins.

**Conclusion:**

Supraphysiologic levels of testosterone caused by testosterone implants as a treatment of hypoactive sexual desire in postmenopausal women can contribute to thrombotic events in the presence of additional prothrombotic risk factors. Therefore, testosterone therapy should include a thorough risk assessment for prothrombotic states, be tailored to patients’ physiologic testosterone levels, and have close follow-up with testosterone level monitoring.

## Background

Portal vein thrombosis (PVT) is a vascular disease that result from mechanical obstruction to the portal vein by a thrombus. Occasionally, the thrombus can extend towards the mesenteric and splenic veins [1–5]. A feared complication is intestinal infarction, which requires prompt surgical exploration [6]. Although uncommon, PVT should be included in the differential diagnosis in the evaluation of postmenopausal women on testosterone replacement therapy (TRT) presenting with acute abdominal pain.

The diagnosis is clinically supported by imaging findings. Ultrasound is the initial examination of choice, with computed tomography (CT) scan providing additional information [3]. PVT most commonly presents in patients with liver disease, malignancy, and also inherited or acquired prothrombotic states [1–5]. Some medications such as those used for hormonal therapy including testosterone have been associated with increased hypercoagulability [7]. TRT has been used on postmenopausal women to increase libido [8, 9]. A 2019 global consensus position statement by an international expert panel recommended against the use of testosterone formulations, including pellets that may cause supraphysiologic levels, because of the possibility of adverse effects, including increased prothrombotic state [10].

Management of PVT consists of investigating possible causes such as liver disease, malignancy, and prothrombotic states. Additionally, treatment with anticoagulation, thrombolysis, or thrombectomy should start as soon as possible to improve outcomes [2–5]. Prompt diagnosis and treatment lead to a favorable prognosis and reduce the risk of acute and chronic complications such as intestinal infarction and portal hypertension [3, 6, 11].

We report a case that describes portomesenteric venous thrombosis in a postmenopausal female on testosterone therapy to treat hypoactive sexual desire. This case demonstrates that testosterone treatment with supraphysiologic levels can facilitate a thrombotic event in the presence of additional prothrombotic risk factors and highlights the importance of its early detection and treatment.

## Case report

A 55-year-old obese postmenopausal Hispanic female, with a body mass index (BMI) of 30.5 kg/m^2^, presented to the emergency department in January 2019 with a 2-day history of diffuse and cramp-like abdominal pain that was worsened by eating. She denied nausea, vomiting, diarrhea, blood in the stool, abdominal trauma, recent surgery, and traveling. Medical history revealed moderate chronic obstructive pulmonary disease (COPD) without a history of recent exacerbation. Medications included Ventolin as needed, Symbicort, Spiriva without any new adjustments, and testosterone pellets implanted 2 months prior for treatment of reduced libido. She denied smoking, alcohol, and drug use. Family history was unremarkable. On physical examination, the patient had oxygen saturation of 95% on ambient air, heart rate 104 beats per minute, blood pressure 135/80 mmHg, and temperature 97.7 °F. Moreover, she exhibited unlabored bilateral breathing with good air entry, and her lungs had bibasilar reduced breath sounds, no chest wall retractions, and no use of accessory muscles.

**Fig. 1:**
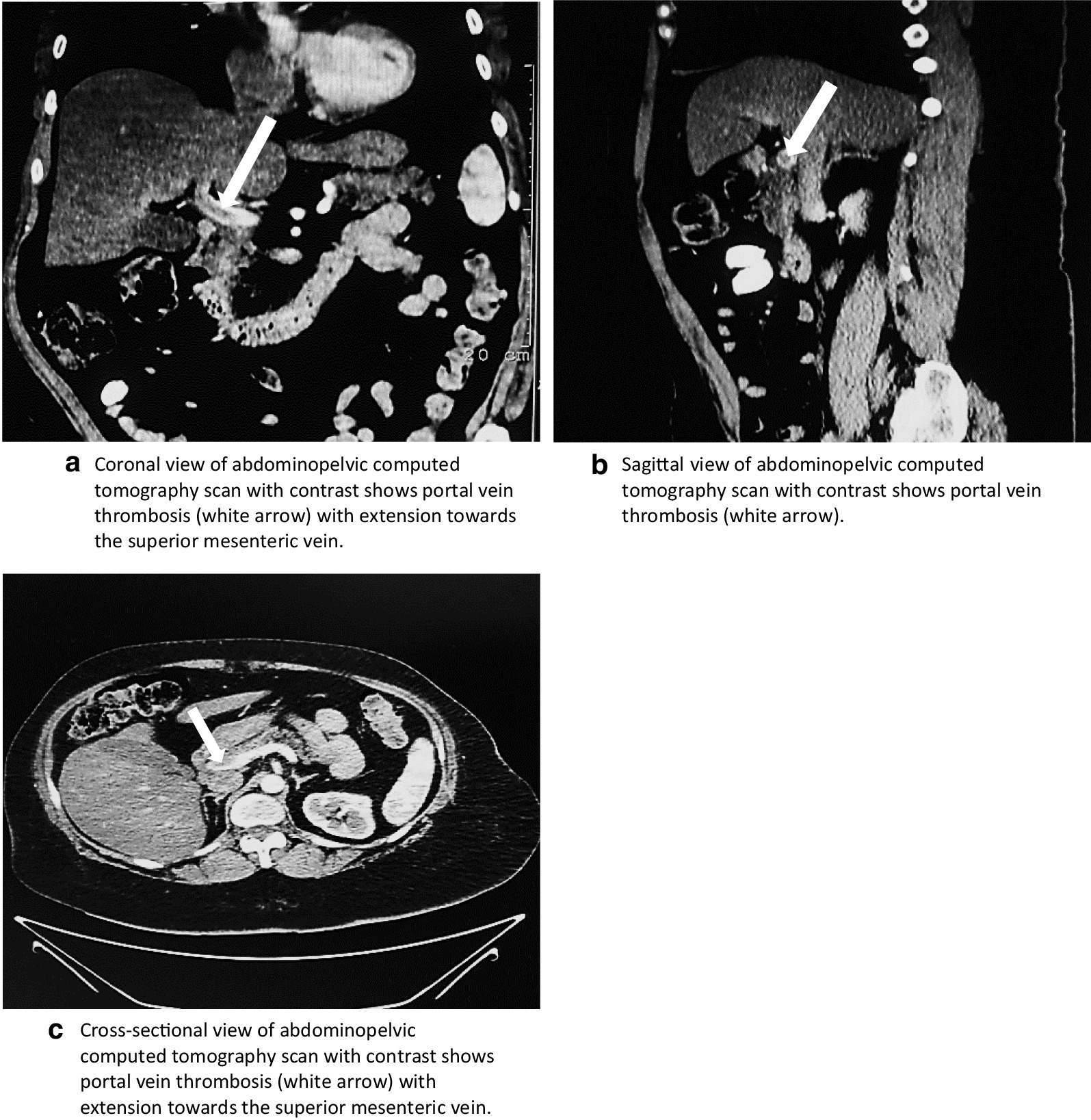
Coronal (**a**), sagittal (**b**) and cross-sectional (**c**) views of abdominopelvic computed tomography scan with contrast demonstrating portal vein thrombosis (white arrows) with extension to the superior mesenteric vein

**Fig. 2:**
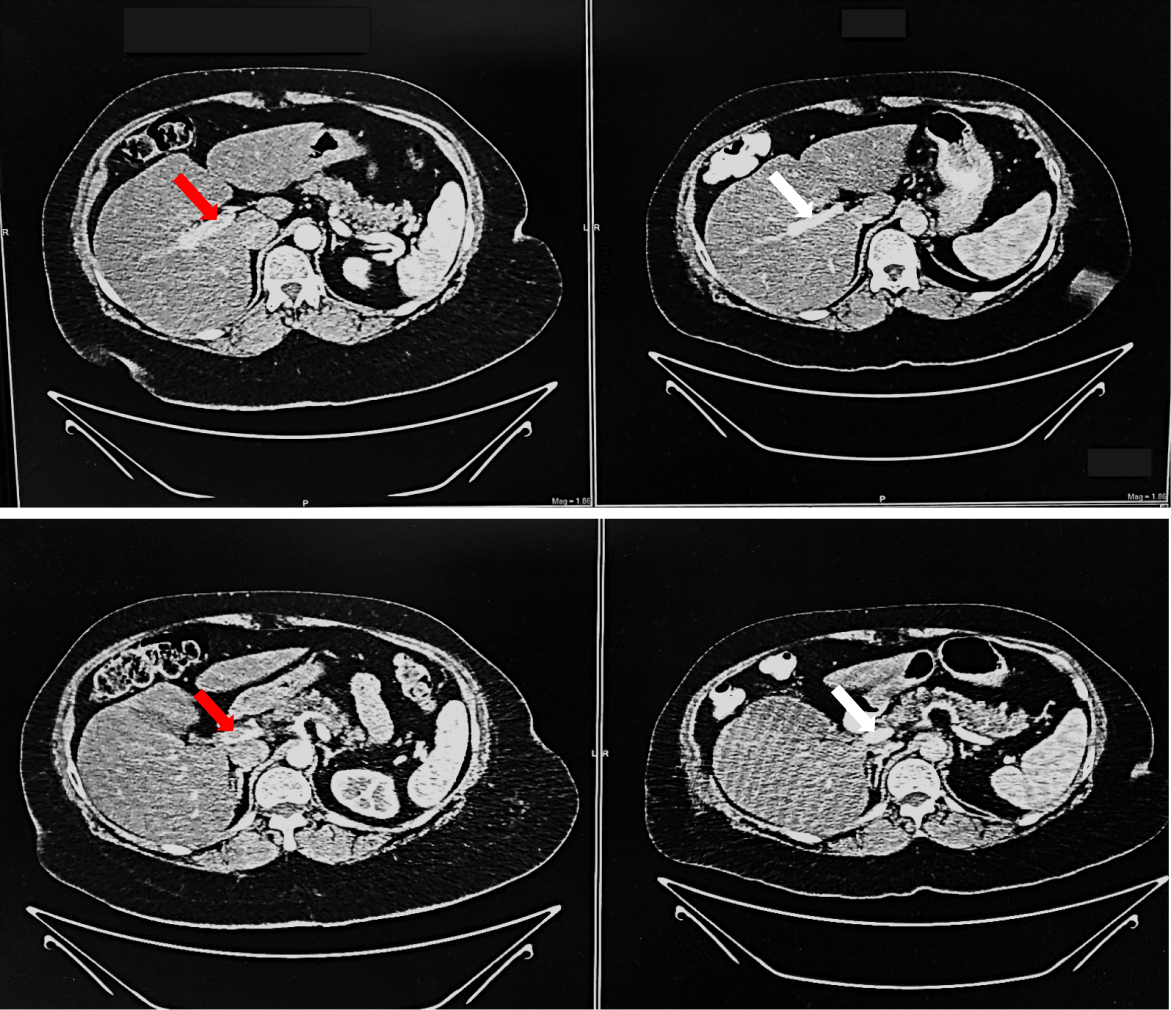
Cross-sectional views of abdominopelvic computed tomography scan with contrast comparing thrombosis of the portal vein (red arrows) before treatment and partial recanalization (white arrows) 3 days after beginning treatment

The abdomen was soft, distended, tender to palpation in the mid-abdomen and right subcostal area, reduced bowel sounds throughout, and no rebound tenderness or guarding. Laboratory studies revealed hemoglobin 17.5 g/dL, hematocrit 52%, leukocytes 9600 per μL, aspartate aminotransferase 55 units per L, alanine aminotransferase 62 units per L, lactic acid 1.4 mmol/L, lipase 98 units per L, a negative fecal occult blood test and basic metabolic panel, urinalysis, and PT/PTT within normal limits. Abdominopelvic CT scan with contrast revealed portal vein thrombosis extending to the mesenteric vein (Fig. 1). Further evaluation excluded known causes of inherited and acquired prothrombotic states. Additionally, testosterone levels were 182 ng/dL. Hematology–oncology suggested a developed prothrombotic state possibly precipitated by TRT, chronic hypoxia, and obesity. Patient treatment consisted of anticoagulation with low-molecular-weight heparin, which gradually improved her abdominal pain. A follow-up abdominal CT scan on day 3 of anticoagulation showed partial portomesenteric vein recanalization (Fig. 2). The patient was later discharged with rivaroxaban for 6 months of anticoagulation therapy and was advised against further HRT. Three months after discharge, she had hemoglobin of 14.5 g/dL and no evidence of prior symptoms or signs of portal hypertension.

## Discussion

PVT is an uncommon medical condition with potentially devastating consequences if not recognized early. It has an annual incidence of 0.7–1:100,000 and a lifetime risk in the general population of 1%. Prevalence is higher in males between 45 and 60 years [1]. PVT is defined as the partial or complete thrombotic occlusion of the portal vein, sometimes with extension towards the mesenteric and/or the splenic veins. It tends to be more frequent in patients with liver disease but is also seen in patients without liver disease and usually associated with an inherited or acquired prothrombotic state. Presentation of disease can be acute or chronic, with portal hypertension being a complication of chronic disease [1–5]. In more than 90% of cases, acute PVT manifests with progressive abdominal pain and distention. Other commonly accompanying symptoms are fever and ascites. In extreme cases, the patient may present signs of acute abdomen and shock indicating bowel infarction [2–4, 6].

The initial diagnostic examination of choice is ultrasound of the portal vein, which can show partial or complete filling of the vein with a solid isoechoic or hypoechoic material. Ultrasound has a sensitivity and specificity range of 80–100%. CT and angiography provide additional information on clot extent and presence of intestinal infarct that helps in the diagnosis with a sensitivity close to 90% [1–3]. Radiological features of acute versus chronic PVT are high luminal density in the thrombosed vessel, lack of porto-portal collaterals, normal spleen size, no myeloproliferative disease, signs of intestinal wall edema, mesenteric vein anomaly, and filling defect [3].

As previously mentioned, in patients without liver cirrhosis or malignancy, PVT may occur due to inherited or acquired prothrombotic states. Inherited prothrombotic states are caused by genetic mutations. These mutations include factor V Leiden, prothrombin G20210A, and deficiencies of proteins C, S, and antithrombin III. Acquired prothrombic states are caused by changes in substances that affect coagulation and may be secondary to immobilization, trauma, surgery, pregnancy, malignancy, and medications. Antiphospholipid antibody syndrome, hyperhomocysteinemia, and myeloproliferative diseases are additional examples [7]. Medications that have been associated with prothrombotic states include hormonal therapies such as oral contraceptive pills, hormone replacement therapy (estradiol, progesterone, and testosterone), and tamoxifen (a selective estrogen receptor modulator). Additionally, anabolic steroids, methotrexate, erythropoietin, corticosteroids, chemotherapeutic agents, and cyclooxygenase 2 inhibitors have also been associated with elevated hypercoagulability [1, 6, 7].

Testosterone has been used for many years to improve libido in postmenopausal women [8, 9]. Testosterone pellets work by releasing small amounts of testosterone in the subcutaneous tissue, presumably over the span of 3–6 months. Testosterone pellets are still not well regulated; for instance, they are very difficult to remove once inserted and can lead to levels higher than those found in premenopausal women, for which women need to be closely monitored. In 2019, a global consensus position statement for the use of testosterone therapy in women diagnosed with hypoactive sexual desire disorder and sexual arousal disorder did not recommend the use of any preparation that results in supraphysiologic concentrations of testosterone, including pellets and injections [10]. However, no significant association with an increased risk of venous thromboembolism has been seen with TRT. The etiology behind this increased risk is still unknown, although it is hypothesized to involve interactions with undiagnosed procoagulable factors [11]. Another possibility is testosterone's known effect stimulating erythropoietin secretion, which leads to increases in hemoglobin, hematocrit, and possibly blood viscosity. Nevertheless, these events represent a low risk of inducing prothrombotic events by themselves unless other risks such as chronic hypoxemia as seen in sleep apnea, obesity hypoventilation, COPD, or smoking coexist [1, 12, 13]. A study conducted to investigate an association between secondary polycythemia and the risk of venous thromboembolism did not show clearly that polycythemia secondary to COPD or other chronic hypoxic conditions independently increases the risk of thrombosis. However, the authors did note that patients in the study with venous thromboembolism had significantly higher BMI [12]. The dangers of venous thromboembolism by testosterone most probably depends on the patient’s age, weight, hormonal status, and additional prothrombotic risk factors.

Management of PVT involves investigating probable causes such as malignancy, liver cirrhosis, hereditary and acquired prothrombotic states, and treatment consisting of anticoagulation with low-molecular-weight heparin, heparin, and Coumadin, or new oral anticoagulants. Additional treatment options are intravenous thrombolysis and thrombectomy [2–5]. Intravenous thrombolysis and thrombectomy can be considered if symptoms began within 12–18 hours before presentation. The treatment goal is to restore the portal vein circulation, thus preventing intestinal infarction and the eventual development of portal hypertension. Prompt treatment is essential because the thrombosed vessel rarely recanalizes spontaneously. Studies have shown favorable outcomes with early anticoagulation showing recanalization in the first week of diagnosis. Recanalization rates were complete in 38.3% and partial in 14% of patients [2–5]. The extent of the thrombosis, the presence of ascites, and prothrombotic states are negative predictors for recanalization. After treatment has begun, close monitoring for symptomatology indicating intestinal infarction is important. Improvement in abdominal pain and the presence of bowel sounds are indicators of good response to treatment. Persistent abdominal pain and bloating despite anticoagulation can indicate intestinal infarction, and prompt surgical exploration may be necessary to reduce mortality [2–6].

Studies have recommended anticoagulation for no less than 6 months in patients diagnosed with PVT with no specific cause identified. Long-term or life-long anticoagulation may be recommended if the patient persists in a hypercoagulable state, has recurrent episodes, or has a personal or family history of deep venous thrombosis [2–5].

The prognosis for PVT is favorable if diagnosed early, and anticoagulation started promptly. The 5-year survival rate in this scenario is more than 85% [3, 11]. Complications are mostly related to either other predisposing conditions, late presentation, or surgery. The most feared complication is intestinal infarction because of its high morbidity and mortality of 20–50% [2, 3, 6].

In the present case, our patient started improving after day 2 of anticoagulation. Repeated abdominal CT revealed signs of portal vein recanalization (Fig. 2). The patient was later discharged on rivaroxaban for 6 months, was closely monitored with no recurrence and no evidence of portal hypertension, and was advised against any further TRT in the future.

## Conclusion

We can conclude that, before starting TRT in postmenopausal women with hypoactive sexual desire, it is essential to do a thorough risk assessment for medical conditions as well as inherited and acquired prothrombotic states that may further increase the risk of venous thromboembolism. In addition, testosterone treatment should be tailored to the female physiologic testosterone levels and include close monitoring of pre- and post-treatment testosterone levels. Hopefully, the benefits and risks of TRT for postmenopausal females will continue to be investigated. Finally, PVT is a rare but serious life-threatening event if not recognized and treated early. Therefore, the suspicion index should be high for clinicians evaluating severe abdominal pain in women on TRT.

## Data Availability

All data generated or analyzed during this study are included in this published article.

## Abbreviations

PVT: Portal vein thrombosis
HRT: Hormone replacement therapy
CT: Computed tomography
TRT: Testosterone replacement therapy
BMI: Body mass index
COPD: Chronic obstructive pulmonary disease
